# Clinical and Radiological Outcomes After Revascularization of Hemorrhagic Moyamoya Disease

**DOI:** 10.3389/fneur.2020.00382

**Published:** 2020-05-07

**Authors:** Kaijiang Kang, Jingjing Lu, Yi Ju, Ruijun Ji, Dandan Wang, Yuan Shen, Lebao Yu, Bin Gao, Dong Zhang, Xingquan Zhao

**Affiliations:** ^1^Department of Neurology, Beijing Tiantan Hospital, Capital Medical University, Beijing, China; ^2^China National Clinical Research Center for Neurological Diseases, Center of Stroke, Beijing Institute for Brain Disorders, Beijing, China; ^3^Department of Neurosurgery, Beijing Tiantan Hospital, Capital Medical University, Beijing, China

**Keywords:** moyamoya disease, moyamoya syndrome, intracranial hemorrhage, revascularization, rebleeding

## Abstract

**Objective:** To evaluate clinical and radiological outcomes after revascularization of hemorrhagic moyamoya disease (MMD).

**Materials and Methods:** We retrospectively collected patients with hemorrhagic MMD who received revascularization from January 2011 to June 2018 at a high-volume stroke center. Rebleeding, ischemic stroke, modified Rankin Scale (mRS) and death after revascularization were used to evaluate long-term clinical outcome. Poor neurological outcome was defined as a mRS>2. The changes of original and revascularization collaterals were used to evaluate radiological outcome. The clinical and radiological outcomes between patients with different surgical revascularization were compared.

**Results:** A total of 312 patients (319 hemispheres) were recruited, including 133 hemispheres (41.7%) with indirect revascularization and 186 hemispheres (58.3%) with direct revascularization. In 308 hemispheres with clinical follow-up data, Postoperative rebleeding, ischemic stroke, poor neurological outcome and death occurred in 13.0% (40/308), 2.6% (8/308), 12.0% (37/308), and 6.2% (19/308) of the hemispheres, respectively. The rates of postoperative rebleeding (8.5 vs. 19.1%, *P* = 0.006) and poor neurological outcome (8.5 vs. 16.8%, *P* = 0.026) were lower in hemispheres with direct revascularization than those with indirect revascularization. However, there was no statistically significant difference in the rates of postoperative ischemic stroke (1.1 vs. 4.6%, *P* = 0.129) and death (4.5 vs. 8.4%, *P* = 0.162) between the two groups. Multivariate logistic regression analysis indicated that the risk of postoperative rebleeding was higher in those with untreated aneurysms, repetitive bleeding episodes, normal perfusion status, and indirect revascularization (*P* < 0.05). In 78 hemispheres with radiological follow-up data, the regression of moyamoya vessels, anterior choroidal artery (AchA), posterior communicating artery (PcomA) and aneurysms were present in 44.9, 47.4, 25.6, and 11.5% of the hemispheres, respectively. The regression of original collaterals and establishment of revascularization collaterals were more significant in hemispheres with direct revascularization than those with indirect revascularization (*P* < 0.05).

**Conclusion:** Direct revascularization may be superior to indirect revascularization for prevention of rebleeding and poor neurological outcome in adults with hemorrhagic MMD. The risk of postoperative rebleeding was higher in those with untreated aneurysms, repetitive bleeding episodes, normal perfusion status, and indirect revascularization. The regression of original collaterals and establishment of revascularization collaterals after revascularization were more significant in hemispheres with direct revascularization than those with indirect revascularization.

## Introduction

Moyamoya disease (MMD) is a relatively rare cerebrovascular disease with unknown etiology, and it is called moyamoya syndrome (MMS) if it is associated with an underlying disease (1–3). With the development and application of non-invasive techniques, an increasing number of MMD have been reported around the world, especially in Japan, Korea, and China (4–9). As the disease progresses, there may be varying degrees of cerebral ischemia or intracranial hemorrhage, and hemorrhagic stroke is one of the main factors leading to death and severe disability. Hemorrhagic MMD is most common in Asian populations, and the proportion of hemorrhagic MMD was up to 21 and 42.4% in the Hokkaido area of Japan and in Korea, respectively (10–12). It is believed that intracranial hemorrhage secondary to MMD occurs mainly owing to the rupture of abnormal moyamoya vessels, dilated collateral vessels, and complicated aneurysms (13–15). Some studies have shown that revascularization surgery could promote the regression of dilated collaterals and complicated aneurysms, and decrease the risk of rebleeding in patients with hemorrhagic MMD (16–18). However, there is still much controversy with respect to the most appropriate therapy and outcomes. In this study, we attempt to evaluate the clinical and radiological outcomes of hemorrhagic MMD after different surgical method of revascularization.

## Materials and Methods

### Study Population

The study was performed according to the guidelines from the Helsinki Declaration, and it was approved by the Institutional Review Board (IRB) of the hospital. All patients or their legally authorized guardian were given written informed consent according to the requirements established by the ethics committee. We collected consecutive patients with hemorrhagic MMD who received revascularization in our hospital from January 2011 to June 2018. Patients who had undergone revascularization surgery before were excluded.

### Baseline Radiological Characteristics

All of the patients underwent digital subtraction angiography (DSA) or magnetic resonance imaging (MRI) in our hospital to confirm the diagnosis of MMD according to the diagnostic guidelines proposed by the Ministry of Health and Welfare of Japan (19). Each hemisphere was analyzed separately. All of the patients with hemorrhagic MMD were confirmed by computed tomography (CT). For bilateral intraventricular hemorrhage (IVH) or subarachnoid hemorrhage (SAH), the side with more hemorrhage was regarded as hemorrhagic hemisphere, and those indistinguishable hemispheres were excluded. According to manifestations of preoperative DSA, all of the hemispheres included were categorized into 6 stages, based on Suzuki stage (20, 21). The dilation and extension of anterior choroidal artery (AchA) and posterior communicating artery (PcomA) were evaluated, and the AchA and PcomA dilation were defined as grade 2 according to the method used in previous studies (13, 22). The preoperative perfusion status was categorized into five stages (Stage 0–4) based on CT perfusion according to a previous study (23): Stage 0, reginal time to peak (rTTP), reginal mean transit time (rMTT), reginal cerebral blood flow (rCBF) and reginal cerebral blood volume (rCBV) were normal; Stage 1, rTTP was delayed, rMTT, rCBF, and rCBV were normal; Stage 2, rTTP and rMTT were delayed, rCBF was normal, and rCBV was normal or slightly increased; Stage 3, rTTP and rMTT were delayed, rCBF was decreased, and rCBV was normal or slightly decreased; Stage 4, rTTP and rMTT were delayed, rCBF and rCBV were decreased. The contralateral mirror area was set as the reference area for unilateral MMD patients, and the cerebellum hemisphere was set as reference area for bilateral MMD patients. Two trained neurologists, who were blinded to the types of revascularization surgery, were responsible for reviewing and assessing all of the radiological variables used in this study and the differences between their assessments were resolved by a third senior neurologist.

### Surgical Revascularization

The operations were performed by experienced neurosurgeons. The neurosurgeons were instructed to choose the appropriate surgical methods based on their experience and preference as well as the conditions of individual patients. The surgical methods included indirect and direct revascularization. Indirect revascularization included encephaloduroarteriosynangiosis (EDAS) and Multiple burr hole trephination. EDAS involves placement of a superficial temporal artery branch (most commonly parietal branch) beneath the dura in ischemic territories. After the detachment of the donor artery with connective tissues (arterial bridge), two burr holes were made beneath the proximal and distal ends of the arterial bridge. The burr holes were connected by milling to make an oval bone flap with a diameter up to 5 cm in major axis, and the dura was fully opened with preservation of middle meningeal artery branches. The arterial bridge was then sutured to the residual dura mater, and the bone flap was finally reset. Multiple burr hole trephination referred to drilling 5 to 8 holes with a diameter of 15 mm in the frontotemporal area. The specific location of drilling depended on the situation of cerebral ischemia and the collateral vessels. After trephination, the dura was opened widely preserving the meningeal arteries. The periosteum above the bone hole was fixed to the surface of the brain through the bone hole and dura matter. Direct revascularization referred to superficial temporal artery to middle cerebral artery (STA-MCA) anastomosis, with or without EDAS. The frontal or parietal branch of superficial temporal artery was dissected and sutured to the M4 segment of middle cerebral artery by end-to-side anastomosis.

### Clinical and Radiological Outcome

Transient ischemic attacks (TIA), ischemic stroke, intracerebral hemorrhage, subdural hemorrhage, intracranial infection, epilepsy, etc. that occurred within 30 days after revascularization surgery were used to evaluate the perioperative safety. Follow-up information on clinical outcome was reviewed by two trained doctors who were blinded to subtypes of revascularization via face-to-face or telephone interview. Intracranial rebleeding, ischemic stroke, modified Rankin Scale (mRS), and death that occurred 30 days after revascularization surgery were used to evaluate the long-term clinical outcome. Poor neurological outcome was defined as a mRS>2.

The radiological outcome was evaluated in those with pre- and postoperative DSA. Postoperative angiographic features included: (1) regression of moyamoya vessels (defined as a reduction in the supplying area by at least a quarter), (2) regression of AchA and PcomA (defined as a reduction in the supplying area or the diameter of initial segment by at least a quarter), (3) regression of complicated aneurysms (defined as disappearance of aneurysms), and (4) development of postoperative revascularization collaterals [according to the system described by Matsushima (24)].

### Statistical Analysis

The statistical analysis was performed using a commercial statistical software package (SPSS for Windows, version 25.0, IBM-SPSS, Chicago, IL, US). Normal distribution data were expressed as mean±SD, while skew distribution data were expressed as median and interquartile range (IQR). The chi-square test and logistic regression were used to analyze the relationship between different treatments and clinical or radiological outcome. The chi-square test (categorical variables), *t*-test (normal distribution), or Mann–Whitney *U*-test (skewed distribution), and logistic regression were used to analyze the differences of clinical and radiological characteristics between hemispheres with or without rebleeding. Kaplan–Meier survival method was used to analyze the relationship between different surgical method and rebleeding. Differences of *P* < 0.05 were considered statistically significant for two-tailed tests.

## Results

### Patient Baseline Characteristics

**Figure 1:**
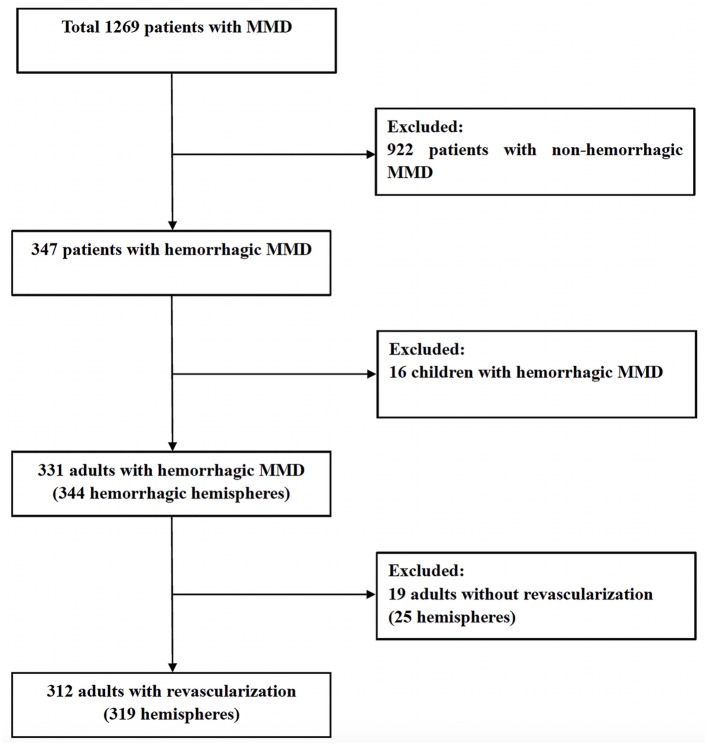
Study flow chart of patient selection.

From January 2011 to June 2018, among 1269 patients with MMD, 319 hemorrhagic hemispheres in 312 adults (39.4 ± 9.1 years old) with hemorrhagic MMD were recruited, including 125 (39.2%) male hemispheres and 194 (60.8%) female hemispheres (Figure 1). The hemorrhagic location included intraventricular hemorrhage (*n* = 127, 39.8%), intracerebral hemorrhage with intraventricular hemorrhage (*n* = 107, 33.5%), intracerebral hemorrhage (*n* = 59, 18.5%) and subarachnoid hemorrhage (*n* = 30, 9.5%). Of these, 280 hemispheres (87.8%) experienced a single episode of hemorrhage, and other 39 hemispheres (12.2%) experienced two or more episodes of hemorrhage. Of the 319 hemorrhagic hemispheres in this study, TIA occurred in 50 hemispheres (15.7%) and ischemic stroke occurred in 24 hemispheres (7.5%) before revascularization surgery. The preoperative CTP suggested that 86.8% (277/319) of the hemispheres were complicated with varying degrees of hypoperfusion (Stage 1–4), while 8.2% (26/319) had no hypoperfusion (Stage 0). The AchA dilatation, PcomA dilatation and combined aneurysm were present in 162 (50.8%), 141 (44.2%) and 43 hemispheres (13.5%), respectively. Of the 319 hemispheres, 133 hemispheres (41.7%) were treated with indirect revascularization (124 hemispheres with EDAS, and 9 hemispheres with multiple burr holes), and 186 hemispheres (58.3%) with direct revascularization (125 hemispheres with STA-MCA anastomosis, and 61 hemispheres with STA-MCA anastomosis combined with EDAS). All baseline characteristics were shown in Table 1.

**Table 1:** Baseline characteristics of 319 hemispheres with hemorrhagic MMD.

**Variable**	**Value**
Male, *n* (%)	125 (39.2%)
Age, mean ±SD	39.4 ± 9.1
Risk factors	
Hypertension, *n* (%)	63 (19.7%)
Diabetes mellitus, *n* (%)	8 (2.5%)
Hyperlipidemia, *n* (%)	5 (1.6%)
Hyperhomocysteinemia, *n* (%)	3 (0.9%)
Smoking, *n* (%)	40 (12.5%)
Location of hemorrhage, *n* (%)	
IVH	127 (39.8%)
ICH+IVH	107 (33.5%)
ICH	55 (17.2%)
SAH	26 (8.2%)
SAH+ICH	4 (1.3%)
Bleeding episodes, *n* (%)	
Once	280 (87.8%)
Repetitive	39 (12.2%)
Ischemic symptoms	
TIA	50 (15.7%)
Ischemic stroke	24 (7.5%)
Perfusion status	
Stage 0	26 (8.2%)
Stage 1	34 (10.7%)
Stage 2	93 (29.2%)
Stage 3	113 (35.4%)
Stage 4	37 (11.6%)
Suzuki Stage, *n* (%)	
Suzuki 1–2	5 (1.6%)
Suzuki 3–4	212 (66.5%)
Suzuki 5–6	50 (15.7%)
AchA dilatation, *n* (%)	162 (50.8%)
PcomA dilatation, *n* (%)	141 (44.2%)
Combined aneurysms, *n* (%)	43 (13.5%)
Revascularization, *n* (%)	
Indirect	133 (41.7%)
Direct	186 (58.3%)
Baseline mRS, median (IQR)	0 (0–2)

*ICH, intracerebral hemorrhage; IVH, intraventricular hemorrhage; SAH, subarachnoid hemorrhage; TIA, transient ischemic attack; AchA, anterior choroidal artery; PcomA, posterior communicating artery; mRS, modified Rankin Scale; IQR, interquartile range*.

### Clinical Outcomes

The perioperative complications included TIA (*n* = 18, 5.6%), ischemic stroke (*n* = 5, 1.6%), intracerebral hemorrhage (*n* = 4, 1.3%), subdural hemorrhage (3, 0.9%), intracranial infection (*n* = 3, 0.9%) and epilepsy (*n* = 3, 0.9%). The incidence of perioperative complications was 12.4% (23/186) in direct revascularization group and 9.0% (12/133) in indirect revascularization group. There was no significant difference in the rates of complications between the hemispheres with direct or indirect revascularization (*p* > 0.05) (Table 2).

**Table 2:** The Perioperative complications in 319 operated hemispheres and long-term clinical outcomes in 308 operated hemispheres with available follow-up data.

	**Total (*n* = 319/308)**	**Indirect (*n* = 133/131)**	**Direct (*n* = 186/177)**	***P*-value**
Perioperative complications (*n* = 319)				
TIA	18 (5.6%)	7 (5.3%)	11 (5.9%)	0.804
Ischemic stroke, *n* (%)	5 (1.6%)	1 (0.8%)	4 (2.2%)	0.593
Intracerebral hemorrhage, *n* (%)	4 (1.3%)	1 (0.8%)	3 (1.6%)	0.864
Subdural hemorrhage	3 (0.9%)	2 (1.5%)	1 (0.5%)	0.769
Intracranial infection	3 (0.9%)	0 (0.0%)	3 (1.6%)	0.377
Epilepsy	3 (0.9%)	1 (0.8%)	2 (1.1%)	>0.999
Long-term outcome (*n* = 308)				
Rebleeding, *n* (%)	40 (13.0%)	25 (19.1%)	15 (8.5%)	0.006
Ischemic stroke, *n* (%)	8 (2.6%)	6 (4.6%)	2 (1.1%)	0.129
mRS>2, *n* (%)	37 (12.0%)	22 (16.8%)	15 (8.5%)	0.026
Death, *n*, (%)	19 (6.2%)	11 (8.4%)	8 (4.5%)	0.162

*TIA, transient ischemic attacks; mRS, modified Rankin Scale*.

**Figure 2:**
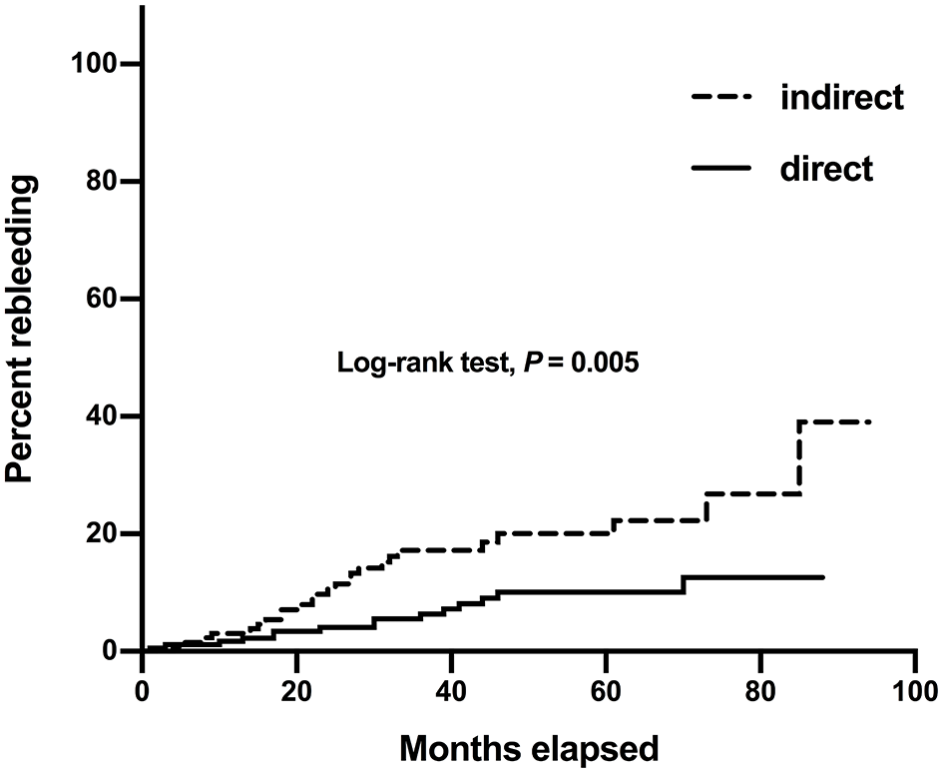
Kaplan–Meier curves for rebleeding incidence in 308 hemorrhagic hemispheres with different revascularization surgery.

Of the 319 hemispheres, 308 hemispheres had clinical follow-up data. The median follow-up duration was 48 (IQR 32–67) months. Postoperative rebleeding occurred in 13.0% (40/308) of the hemispheres, of which 13 hemispheres (32.5%) had died and 14 hemispheres (35.0%) had been severely disabled (mRS>2). The median interval from revascularization to subsequent rebleeding was 24.5 months (IQR 15.3–38.3). The rate of postoperative rebleeding was lower in hemispheres with direct revascularization than those with indirect revascularization (8.5 vs. 19.1%, *P* = 0.006) (Table 2). After adjusted by age, gender, hypertension, bleeding location, bleeding episodes, Suzuki stage, AchA dilatation, PcomA dilatation, untreated aneurysms, and perfusion status, direct revascularization was still associated with lower risk of postoperative rebleeding, compared with indirect revascularization (*P* = 0.032, OR = 0.419) (Table 3). Kaplan-Meier curve for rebleeding incidence also demonstrated that the probability of rebleeding was lower in those who received direct revascularization than those who received indirect revascularization. The Log-rank test revealed that there was a significant difference between the two survival curves (*P* = 0.005) (Figure 2). The annual rebleeding rate of overall, direct group, and indirect group were 2.3, 1.7, and 3.1%, respectively.

**Table 3:** Relationship between subtypes of revascularization and rebleeding.

**Model**	***P*-value**	**OR**	**95 % CI**	
			**Lower**	**Upper**
Model 1	0.007	0.393	0.198	0.779
Model 2	0.007	0.393	0.198	0.779
Model 3	0.007	0.393	0.198	0.779
Model 4	0.004	0.350	0.173	0.711
Model 5	0.005	0.344	0.164	0.724
Model 6	0.014	0.385	0.180	0.824
Model 7	0.032	0.419	0.189	0.930

*Model 1: univariate analysis of revascularization subtypes*.

*Model 2: adjusted by age and gender on the basis of Model 1*.

*Model 3: adjusted by hypertension on the basis of Model 2*.

*Model 4: adjusted by bleeding location and bleeding episodes on the basis of Model 3*.

*Model 5: adjusted by the Suzuki stage on the basis of Model 4*.

*Model 6: adjusted by the AchA dilatation, PcomA dilatation and untreated aneurysms on the basis of Model 5*.

*Model 7: adjusted by the perfusion status on the basis of Model 6*.

The proportions of untreated aneurysms (25.6 vs. 11.6%, *P* = 0.019), repetitive bleeding episodes (22.5 vs. 10.8%, *P* = 0.036), normal perfusion status (25.6 vs. 5.9%, *P* < 0.001), and indirect revascularization (62.5 vs. 39.6%, *P* = 0.006) in hemispheres with rebleeding were significantly higher than those without rebleeding. Multivariate logistic regression analysis indicated that the risk of postoperative rebleeding was higher in those with untreated aneurysms, repetitive bleeding episodes, normal perfusion status, and indirect revascularization (*P* < 0.05). The differences of baseline characteristics between hemispheres with or without rebleeding were shown in Table 4. Among hemispheres only with direct or indirect revascularization, the proportions of untreated aneurysms, repetitive bleeding episodes, normal perfusion status in hemispheres with rebleeding were also relatively higher than those without rebleeding, but without sustained statistical difference (Table 5).

**Table 4:** Differences in clinical and neuroradiological characterizations between hemispheres with or without rebleeding.

	**Rebleeding (*n* = 40)**	**Non-rebleeding (*n* = 268)**	***P_**1**_* value (univariate)**	***P_**2**_* value (multivariate)**
Male, *n* (%)	13 (32.5%)	108 (40.3%)	0.346	0.909
Age, mean ±SD	40.6 ± 6.9	39.2 ± 9.3	0.389	0.565
Hypertension, *n* (%)	11 (27.5%)	50 (18.7%)	0.191	0.080
Diabetes mellitus, *n* (%)	0 (0.0%)	7 (2.6%)	0.642	0.401
Hyperlipidemia, *n* (%)	1 (2.5%)	4 (1.5%)	>0.999	0.714
Smoking, *n* (%)	5 (12.5%)	34 (12.7%)	0.974	0.952
Suzuki Stage, *n* (%)			0.131	0.274
Suzuki 1–2	0 (0.0%)	5 (2.3%)		
Suzuki 3–4	26 (70.3%)	177 (80.8%)		
Suzuki 5–6	11 (29.7%)	37 (16.9%)		
AchA dilatation, *n* (%)	21 (55.3%)	134 (61.2%)	0.491	0.209
PcomA dilatation, *n* (%)	19 (50.0%)	116 (53.0%)	0.735	0.520
Untreated aneurysms, *n* (%)	10 (25.6%)	26 (11.6%)	0.019	0.011
Location of hemorrhage, *n* (%)			0.159	0.762
IVH	18 (45.0%)	100 (37.3%)		
IVH+ICH	15 (37.5%)	90 (33.6%)		
ICH	7 (17.5%)	48 (17.9%)		
SAH/SAH+ICH	0 (0.0%)	30 (11.2%)		
Bleeding episodes, *n* (%)			0.036	0.019
Once	31 (77.5%)	239 (89.2%)		
Repetitive	9 (22.5%)	29 (10.8%)		
Perfusion status			<0.001	<0.001
Stage 0	10 (25.6%)	15 (5.9%)		
Stage 1–4	29 (74.4%)	238 (94.1%)		
Revascularization, *n* (%)			0.006	0.032
Indirect	25 (62.5%)	106 (39.6%)		
Direct	15 (37.5%)	162 (60.4%)		

*AchA, anterior choroidal artery; PcomA, posterior communicating artery; ICH, intracerebral hemorrhage; IVH, intraventricular hemorrhage; SAH, subarachnoid hemorrhage; Chi-square test and logistic regression were used to analyze the differences of clinical and neuroradiological characterizations between rebleeding hemispheres and non-rebleeding hemispheres*.

**Table 5:** Stratified analysis of differences in clinical and neuroradiological characterizations between hemispheres with or without rebleeding among hemispheres only with direct or indirect revascularization.

	**Direct revascularization**			**Indirect revascularization**		
	**Rebleeding (*n* = 15)**	**Non-rebleeding (*n* = 162)**	***P*-value**	**Rebleeding (*n* = 25)**	**Non-rebleeding (*n* = 106)**	***P*-value**
Male, *n* (%)	10 (66.7%)	66 (40.7%)	0.052	3 (12.0%)	42 (39.6%)	0.009
Age, mean ±SD	36.7 ± 7.2	39.7 ± 9.5	0.229	42.9 ± 5.6	38.5 ± 9.0	0.003
Hypertension, *n* (%)	5 (33.3%)	34 (21.0%)	0.437	6 (24.0%)	16 (15.1%)	0.439
Diabetes mellitus, *n* (%)	0 (0.0%)	5 (3.1%)	>0.999	0 (0.0%)	2 (1.9%)	>0.999
Hyperlipidemia, *n* (%)	1 (6.7%)	3 (1.9%)	0.301	0 (0.0%)	1 (0.9%)	>0.999
Smoking, *n* (%)	3 (20.0%)	20 (12.3%)	0.658	2 (8.0%)	14 (13.2%)	0.707
Suzuki Stage, *n* (%)			0.277			0.585
Suzuki 1–2	0 (0.0%)	3 (2.2%)		0 (0.0%)	2 (2.4%)	
Suzuki 3–4	10 (71.4%)	114 (84.4%)		16 (69.6%)	63 (75.0%)	
Suzuki 5–6	4 (28.6%)	18 (13.3%)		7 (30.4%)	19 (22.6%)	
AchA dilatation, *n* (%)	8 (57.1%)	82 (60.7%)	0.793	13 (54.2%)	52 (61.9%)	0.495
PcomA dilatation, *n* (%)	5 (35.7%)	79 (58.5%)	0.101	14 (58.3%)	37 (44.0%)	0.216
Untreated aneurysms, *n* (%)	4 (26.7%)	15 (19.9%)	0.177	6 (25.0%)	11 (12.8%)	0.253
Location of hemorrhage, *n* (%)			0.514			0.161
IVH	7 (46.7%)	62 (38.3%)		11 (44.0%)	38 (35.8%)	
IVH+ICH	5 (33.3%)	61 (37.7%)		10 (40.0%)	29 (27.4%)	
ICH	3 (20.0%)	22 (13.6%)		4 (16.0%)	26 (24.5%)	
SAH/SAH+ICH	0 (0.0%)	17 (10.5%)		0 (0.0%)	13 (12.3%)	
Perfusion status			0.124			<0.001
Stage 0	5 (20.0%)	7 (7.2%)		5 (35.7%)	8 (5.1%)	
Stage 1–4	20 (80.0%)	90 (92.8%)		9 (64.3%)	148 (94.9%)	
Bleeding episodes, *n* (%)			0.957			0.131
Once	13 (86.7%)	147 (90.7%)		18 (72.0%)	92 (86.8%)	
Repetitive	2 (13.3%)	15 (9.3%)		7 (28.0%)	14 (13.2%)	

*AchA, anterior choroidal artery; PcomA, posterior communicating artery; ICH, intracerebral hemorrhage; IVH, intraventricular hemorrhage; SAH, subarachnoid hemorrhage; Chi-square test and logistic regression were used to analyze the differences of clinical and neuroradiological characterizations between rebleeding hemispheres and non-rebleeding hemispheres*.

Postoperative ischemic stroke, poor neurological outcome and death occurred in 2.6% (8/308), 12.0% (37/308) and 6.2% (19/308) of the hemispheres. The rate of poor neurological outcome was lower in hemispheres with direct revascularization than those with indirect revascularization (8.5 vs. 16.8%, *P* = 0.026). However, there was no statistically significant difference in the risk of postoperative ischemic stroke (1.1 vs. 4.6%, *P* = 0.129) and death (4.5 vs. 8.4%, *P* = 0.162) between the two groups (Table 2).

In this study, 17 MMD patients (19 hemorrhagic hemispheres) died and all died of intracranial rebleeding, of which 13 hemispheres died of ipsilateral rebleeding, while the other six hemispheres died of contralateral rebleeding. In addition, 34 MMD patients (37 hemorrhagic hemispheres) had a poor neurological outcome, of which 33 hemispheres were due to intracranial rebleeding and four hemispheres were due to poor baseline neurological function.

### Radiological Outcomes

**Figure 3:**
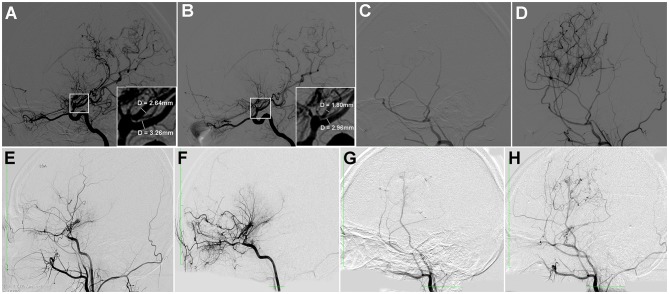
Two cases of hemorrhagic MMD with direct or indirect revascularization. **(A–D)** A 20-year-old female patient with hemorrhagic MMD, who received direct revascularization. Preoperative DSA showed dilation of AchA and PcomA **(A)**. Postoperative DSA a year later showed significant regression of dilated AchA and PcomA **(B)**, with substantial revascularization collaterals **(C,D)**. She was followed-up for 56 months without rebleeding. **(E–H)** A 19-year-old female patient with hemorrhagic MMD, who received indirect revascularization. Preoperative DSA showed mild dilatation of AchA **(E)**. Postoperative DSA a year later showed that the regression of dilatated AchA was not significant **(F)**, with moderate revascularization collaterals **(G,H)**. She was followed-up for 48 months without rebleeding.

Pre- and postoperative DSA were available in 78 hemorrhagic hemispheres. The median of time interval from revascularization surgery to postoperative DSA was 8.1 (IQR 6.5–12.5) months. Of the 78 hemispheres, the regression of moyamoya vessels, AchA, PcomA and aneurysms were present in 44.9% (35/78), 47.4% (37/78), 25.6% (20/78) and 11.5% (9/78) of the hemispheres, respectively. The regression of moyamoya vessels (64.3 vs. 22.2%, *P* < 0.001), AchA (71.4 vs. 19.4%, *P* < 0.001) and PcomA (35.7 vs. 13.9%, *P* = 0.028) were more significant in hemispheres with direct revascularization than those with indirect revascularization. Also, the proportion of substantial revascularization collaterals (grade A) in hemispheres with direct revascularization was significantly higher than those with indirect revascularization (57.1 vs. 16.7%, *P* < 0.001) (Table 6, Figure 3). In addition, we also made a comparison of regression of moyamoya vessel, AChA, PComA and aneurysm between hemispheres with or without rebleeding. The results indicated that the regression proportion of moyamoya vessels (50.0 vs. 21.4%, *P* = 0.052), AchA (51.6 vs. 28.6%, *P* = 0.119) and PcomA (29.7 vs. 7.1%, *P* = 0.080) were relatively higher in hemispheres without rebleeding than those with rebleeding, but without statistically significant difference (Table 6). There was no significant difference in proportion of aneurysm regression and revascularization collaterals between rebleeding hemispheres and non-rebleeding hemispheres (*P* > 0.05) (Table 6).

**Table 6:** The radiological outcomes in 78 operated patients with available follow-up data.

	**Total (*n* = 78)**	**Revascularization**			**Outcome**		
		**Indirect (*n* = 36)**	**Direct (*n* = 42)**	***P*-value**	**Rebleeding (*n* = 14)**	**Non-rebleeding (*n* = 64)**	***P*-value**
Moyamoya vessels regression, *n* (%)	35 (44.9%)	8 (22.2%)	27 (64.3%)	<0.001	3 (21.4%)	32 (50.0%)	0.052
AchA regression, *n* (%)	37 (47.4%)	7 (19.4%)	30 (71.4%)	<0.001	4 (28.6%)	33 (51.6%)	0.119
PcomA regression, *n* (%)	20 (25.6%)	5 (13.9%)	15 (35.7%)	0.028	1 (7.1%)	19 (29.7%)	0.080
Aneurysm regression (n, %)	9 (11.5%)	5 (13.9%)	4 (9.5%)	0.806	2 (14.3%)	7 (10.9%)	>0.999
Matsushima grading				0.001			0.782
A	30 (38.5%)	6 (16.7%)	24 (57.1%)		5 (35.7%)	25 (39.1%)	
B	26 (33.3%)	18 (50.0%)	8 (19.0%)		4 (28.6%)	22 (34.4%)	
C	22 (28.2%)	12 (33.3%)	10 (23.8%)		5 (35.7%)	17 (26.6%)	

*Regression of moyamoya vessels was defined as a reduction in the supplying area by at least a quarter. Regression of AchA and PcomA was defined as a reduction in the supplying area or the diameter of initial segment by at least a quarter. Regression of aneurysms was defined as disappearance of aneurysms. Matsushima grading: A, area perfused by postoperative revascularization collaterals more than 2/3 of the middle cerebral artery (MCA) distribution; B, between 1/3 and 2/3 of the MCA distribution; C, less than 1/3 of the MCA distribution. Chi-square test was used to analyze the differences of neuroradiological characterizations after revascularization surgery*.

## Discussions

In this study, we found that for adults with hemorrhagic MMD, direct revascularization may be superior to indirect revascularization for prevention of rebleeding and poor neurological outcome. The risk of postoperative rebleeding was higher in those with untreated aneurysms, repetitive bleeding episodes, normal perfusion status, and indirect revascularization. The regression of original collaterals and establishment of revascularization collaterals after revascularization were more significant in hemispheres with direct revascularization than those with indirect revascularization.

Due to the unknown etiology and pathogenesis of MMD, there has been no effective conservative treatment, especially the hemorrhagic MMD. Currently, it is generally recognized that revascularization surgery can improve cerebral ischemia symptoms in MMD patients, supplying blood to ischemic brain tissues by collateral vessels (1, 2, 19). A recent propensity-score matched analysis on the difference between indirect and direct bypass in preventing stroke in ischemic MMD showed that direct revascularization provided longer stroke-free survival (25). Hemorrhagic stroke is one of the main factors leading to worse prognoses, such as death and severe disability. It is suggested that intracranial hemorrhage occurs mainly owing to the rupture of abnormal moyamoya vessels, dilated collateral vessels, and complicated aneurysms (13–15). Revascularization surgery was supposed to decrease the intravascular pressure load of original collaterals in internal carotid artery (ICA) system and alleviate the dilation of collateral vessels or complicated aneurysms, which may contribute to decrease the risk of rebleeding in patients with hemorrhagic MMD (13, 17, 18, 26–28). However, there has been much controversy with respect to the most appropriate surgical method and outcome of hemorrhagic MMD.

Although vessel remodeling of the original collaterals has been observed in MMD, there has been much controversy with respect to the outcomes of patients with hemorrhagic MMD who received revascularization surgery (29, 30). The Japan Adult Moyamoya Trial concluded that direct revascularization (extra-intracranial bypass) can reduce the incidence of rebleeding (from 7.6%/y to 2.7%/y) and improve patient prognosis, however, indirect revascularization was not involved in the study, and the result of it was statistically marginal (31). Wang et al. (16) reported that indirect revascularization (Encephaloduroarteriosynangiosis, EDAS) appeared to be also beneficial for patients with hemorrhagic MMD, decreasing the annual rebleeding rate to 2.2%/person. Some studies showed that the rebleeding ratio in patients who received direct revascularization was lower than that in patients treated with indirect revascularization (0 vs. 28.5%) (17). A recent prospective study showed that combined revascularization surgery can help prevent ipsilateral rebleeding in adults with hemorrhagic MMD, decreasing the annual rebleeding rate to 0.62%/person (18). After all, which surgical method of revascularization is most effective remains still controversial.

The postoperative annual rebleeding rate in this study was significantly lower than natural annual rebleeding rate according to a recently published study about the natural history of hemorrhagic MMD (2.3%/person vs. 4.5%/person) (32). Of the 78 hemorrhagic hemispheres with pre- and postoperative DSA data, the regression of moyamoya vessels, dilated AchA or PcomA, and complicated aneurysms were present after revascularization surgery. These changes of radiological characteristics may contribute to the decreased risk of rebleeding after revascularization, according to some previous studies (17, 18, 33).

Compared with indirect revascularization, direct revascularization decreased the risk of postoperative rebleeding and poor neurological outcome. Radiological results also demonstrated that the regression of moyamoya vessels, dilated AchA, and PcomA were more significant in hemispheres with direct revascularization than those with indirect revascularization, and the proportion of substantial revascularization collaterals in hemispheres with direct revascularization was significantly higher than those with indirect revascularization, which can explain in part why direct revascularization decreased the risk of postoperative rebleeding compared with indirect revascularization in this study. Therefore, from the results of this study, the direct revascularization may be superior to indirect revascularization for prevention of rebleeding and poor neurological outcome in adults with hemorrhagic MMD.

Potential limitations of our studies should be mentioned. First, all of the patients in this study were enrolled from a single center, and there were missing values in the radiological analysis and postoperative DSA were performed at varying intervals, so potential selection bias may be inevitable. We, however, tried our best to reduce in-house selection bias by collecting patients consecutively. Second, the surgical procedures that the patients received were not randomized, but based mainly on the surgeons' expertise. Third, the retrospective nature of the study necessitates further prospective cohorts or randomized studies to confirm our conclusions.

## Conclusions

Direct revascularization may be superior to indirect revascularization for prevention of rebleeding and poor neurological outcome in adults with hemorrhagic MMD. The risk of postoperative rebleeding was higher in those with untreated aneurysms, repetitive bleeding episodes, normal perfusion status, and indirect revascularization. The regression of original collaterals and establishment of revascularization collaterals after revascularization were more significant in hemispheres with direct revascularization than those with indirect revascularization.

## Data Availability Statement

The data supporting the conclusions of this article will be made available by the authors, without undue reservation, to any qualified researcher.

## Ethics Statement

The studies involving human participants were reviewed and approved by Institutional Review Board (IRB) of Beijing Tiantan Hospital. The patients/participants provided their written informed consent to participate in this study.

## Author Contributions

KK, XZ, and DZ contributed to the study concept and design, data analysis and interpretation, guarantors of this work, and had full access to the data. KK, JL, and YJ managed the literature searches and analyses. KK undertook the statistical analysis and wrote the first draft of the manuscript. YS, LY, and BG collected and researched data. KK, XZ, DZ, RJ, and DW contributed to critical revision of the manuscript and final approval of the version to be published.

## Conflict of Interest

The authors declare that the research was conducted in the absence of any commercial or financial relationships that could be construed as a potential conflict of interest.
